# *Lactococcus lactis* Mutants Obtained From Laboratory Evolution Showed Elevated Vitamin K2 Content and Enhanced Resistance to Oxidative Stress

**DOI:** 10.3389/fmicb.2021.746770

**Published:** 2021-10-14

**Authors:** Yue Liu, Anteun de Groot, Sjef Boeren, Tjakko Abee, Eddy J. Smid

**Affiliations:** ^1^Food Microbiology, Wageningen University and Research, Wageningen, Netherlands; ^2^Laboratory of Biochemistry, Wageningen University and Research, Wageningen, Netherlands

**Keywords:** adaptive laboratory evolution, lactic acid bacteria, vitamin enrichment, food fermentation, non-GM approach

## Abstract

Vitamin K2 is an important vitamin for human health. Vitamin K2 enrichment in the human diet is possible by using vitamin K2-producing bacteria such as *Lactococcus lactis* in food fermentations. Based on previous observations that aerated cultivation conditions improved vitamin K2 content in *L. lactis*, we performed laboratory evolution on *L. lactis* MG1363 by cultivating this strain in a shake flask in a sequential propagation regime with transfers to a fresh medium every 72h. After 100 generations of propagation, we selected three evolved strains that showed improved stationary phase survival in oxygenated conditions. In comparison to the original strain MG1363, the evolved strains showed 50–110% increased vitamin K2 content and exhibited high resistance against hydrogen peroxide-induced oxidative stress. Genome sequencing of the evolved strains revealed common mutations in the genes *ldh* and *gapB*. Proteomics analysis revealed overproduction of glyceraldehyde 3-phosphate dehydrogenase (GapA), universal stress protein A2 (UspA2), and formamidopyrimidine-DNA glycosylase (MutM) under aerated conditions in evolved strains, proteins with putative functions in redox reactions, universal stress response, and DNA damage repair, all of which could contribute to the enhanced oxidative stress resistance. The mechanisms underlying elevated vitamin K2 content in the evolved strains remain to be elucidated. Two out of the three evolved strains performed similar to the original strain MG1363 in terms of growth and acidification of culture media. In conclusion, this study demonstrated a natural selection approach without genetic manipulations to obtain vitamin K2 overproducers that are highly relevant for food applications and contributed to the understanding of oxidative stress resistance in *L. lactis*.

## Introduction

Vitamin K2 is an essential vitamin for human health. It functions as an enzyme co-factor for the carboxylation of proteins with γ-carboxyglutamic domains (Gla-proteins) that are involved in biological processes such as blood coagulation, calcium metabolism, and cell growth (Vermeer and Schurgers, 2000; Vermeer, 2012). In addition, a higher intake of vitamin K2 was found to be associated with a reduced risk of coronary heart disease and improved bone health (Geleijnse et al., 2004; Plaza and Lamson, 2005; Beulens et al., 2013; Myneni and Mezey, 2018).

Vitamin K2, also referred to as menaquinones, is a group of compounds sharing a naphthoquinone ring structure but differing in the number of the isoprene units in the side chain (Vermeer and Schurgers, 2000). The specific form is expressed as MK-n where n depicts the number of the side chain isoprene units. Vitamin K2 is produced by bacteria, and *Lactococcus lactis* is one of the producers (Morishita et al., 1999; Liu et al., 2019). *Lactococcus lactis* produces mainly MK-9 and MK-8, the long-chain MK forms that are shown to have higher contributions to the vitamin K2 status in human body (Schurgers and Vermeer, 2002; Geleijnse et al., 2004; Schurgers et al., 2007). This is of great interest, as *L. lactis* is widely applied in various food fermentation or biotechnological production processes (Song et al., 2017), allowing opportunities for vitamin K2 enrichment in food products or supplements.

The possibility to obtain vitamin K2 overproducers by genetic engineering has been demonstrated in *L. lactis* (Bøe and Holo, 2020), but practical applications could be limited due to the strict rules in the European Union regarding the use of genetically modified organisms (GMOs; Sybesma et al., 2006; Eriksson et al., 2020). Therefore, an approach without genetic modification (non-GM) is preferred to obtain vitamin K2 overproducers. In this regard, some studies employed an MK analog to select for vitamin K2 overproducers of *Bacillus subtilis*, and these studies reported 30–100% increased production (Sato et al., 2001; Tsukamoto et al., 2001). Efforts have also been made to reveal the cultivation conditions that improve vitamin K2 content in bacteria: For *L. lactis*, Liu et al. (2019) observed that aerobic fermentation increased vitamin K2 content compared to static fermentation.

It is known that menaquinones function as electron carriers in the respiratory electron transport chain (ETC) in the cytoplasmic membranes of the producing bacteria. In *L. lactis*, it has been shown that menaquinones, together with an NADH dehydrogenase complex and the bd-type cytochrome complex, form a simple ETC that enables aerobic respiration when heme (co-factor of cytochrome) and oxygen are supplemented (Rezaïki et al., 2008; Brooijmans et al., 2009). However, not much is known about the roles of menaquinones in *L. lactis* under aerobic (fermentation) conditions without heme-induced respiration. Evidence has been provided in some bacteria that quinones contribute to defense against oxidative stress (Søballe and Poole, 2000; Maruyama et al., 2003; Wang and Maier, 2004). The same protective effect was suggested, although not experimentally confirmed, for menaquinone in *L. lactis* (Vido et al., 2005), where menaquinone is the sole quinone form produced even under non-respiration conditions (Morishita et al., 1999). Although the exact mechanism is not yet revealed, combining these suggestions and the findings by Liu et al. (2019) where vitamin K2 content is improved under aerobic fermentation, we hypothesized that applying aerated conditions could lead to the selection of natural vitamin K2 overproducers.

In this study, laboratory evolution under aerated conditions was performed on *L. lactis* ssp. *cremoris* model strain MG1363, aiming to explore the possibility of an non-GM approach to obtain vitamin K2 overproducers. After propagating approximately hundred generations under intensively aerated conditions, we examined three obtained evolved strains closely for their vitamin K2 (menaquinone) content and other physiological characteristics, as well as the genetic and proteome changes compared to the original strain. Relevant test conditions employed in this study were static fermentation (ST), aerated (AE), and respiration-permissive (RES) conditions.

## Materials and Methods

### Strains and Conditions

*Lactococcus lactis* ssp. *cremoris* MG1363 and evolved strains were cultivated in M17 medium (Oxoid™) supplemented with 0.5% glucose (w/v; named GM17) at 30°C. For static fermentation conditions (ST), the cultures were statically incubated in closed, full filled tubes. For aerated conditions (AE), the cultures were incubated in Erlenmeyer flasks filled with 10% volume media, shaking at 200rpm. For respiration-permissive condition (RES), 2μg/ml heme was added to the cultures besides the settings of the aerated conditions.

### Laboratory Evolution

The evolution was carried out in 100-ml flasks filled with 10ml GM17 media, shaking at 200rpm at 30°C. The first culture was obtained by inoculating a colony of strain MG1363 in the medium. Thereafter every 72h, a passage was made by transferring 1/100 volume of the old culture into fresh medium in a new 100-ml flask. In total 14 passages were made before evolved strains were isolated for analysis. The cultures from the 14th passage were plated, and single colonies were purified. In total two independent cultures (A and B) were successfully maintained throughout all the passages, with nine mutants screened in culture A and five mutants in culture B for vitamin K2 content (specific concentration) under static fermentation conditions. Five out of nine mutants from culture A and all five mutants from culture B showed similar (no increase or less than 30% increase) vitamin K2 content as the wild-type strain, and we selected all three mutants (Evo1 – 3) from culture A showing significant increase in vitamin K2 content for further comparative analysis with the wild-type strain.

### Determination of Bacterial Growth and Survival

To obtain growth curves, overnight cultures of each strain were diluted to OD_600_=0.1 (600nm; path length 10mm) in 50ml fresh GM17 and subjected to desired cultivation conditions (ST, AE or RES). Samples were taken every half hour for optical density (OD) measurement at 600nm by a spectrophotometer. To determine the survival of bacteria, viable plate counts were determined at indicated time points.

### H_2_O_2_ Treatment

An overnight culture of each strain, obtained from static cultivation, was diluted to OD_600_=0.2 with fresh GM17 in a 15-ml centrifugal tube and incubated at 30°C for 1h statically. Then, 5mM hydrogen peroxide was added (*t*=0) and the viable plate count of bacteria was determined every 30min for 2h in total. To stop the action of hydrogen peroxide, 2μl catalase (1.3*10^6^ u/ml) was added to the sample that was examined.

### Acidification Test

An overnight culture of each strain, obtained from static cultivation, was diluted to OD_600_=0.1 with fresh GM17 in a 50-ml centrifugal tube and then incubated statically at 30°C. Thereafter, the pH of the culture was measured by a pH probe every hour, for 6h in total.

### Vitamin K2 Extraction and Analysis

*Lactococcus lactis* cells were cultivated in GM17 media at 30°C for 48h under static fermentation conditions and harvested for vitamin K2 analysis. Vitamin K2 was extracted from cells as described by Liu et al. (2019). Briefly, PBS washed biomass was incubated with 10mg/ml lysozyme (Sigma) at 37°C for 1h. Four volumes of extraction buffer [n-hexane and 2-propanol at a ratio of 2:1 (v/v)] was added to the lysozyme-treated bacterial suspension and vigorously mixed by vortexing. After centrifugation at 3,000×*g* for 10min, the (upper) organic phase was collected. Equal volume (as extraction buffer) of n-hexane was added to the remaining lower phase, and the extraction step was repeated twice. The organic phases from each sample were combined and evaporated under a flow of N_2_ gas, and re-dissolved in iso-propanol. All samples were diluted in methanol and subjected to liquid chromatography–mass spectrometry (LC–MS) analysis for identification and quantification of different forms of vitamin K2.

Samples from two experiments were analyzed exactly as described in a previous study (Liu et al., 2019) by a high-performance liquid chromatography (HPLC; UFLC, Shimadzu, Japan) system coupled with a Micro-mass Quattro Ultima MS (Waters, United States). Samples from another two experiments were analyzed using a UPLC (Thermo Scientific Vanquish) coupled with MS (Thermo Q-Exactive hybrid quadrupole-Orbitrap) as follows: 1μl sample was injected into an Acquity BEH C18 column (50mm×2.1mm, 1.7μm particle, 130A, Waters), and compounds were eluted with a gradient starting from 100% methanol to methanol/isopropanol (75%/25%) in 13.7min and maintained for 5.5min before going back to 100% methanol in 1.1min. The flow rate was 0.4ml/min, and column compartment temperature was kept at 40°C. The MS system was equipped with a heated electrospray ionization (ESI) source that was set at positive ionization mode. The capillary temperature was 150°C, and the source temperature was 450°C. The sheath gas (nitrogen) was set to 40 arbitrary units, and the ion spray voltage was 4.5kV. Data were processed using software Xcalibur (Thermo Scientific, version 2.2).

Analytical standards containing MK-1 (Santa Cruz Biotechnology), MK-4 (Sigma), MK-7 (Sigma), MK-9 (Santa Cruz Biotechnology), and vitamin K1 (Sigma) were mixed in the concentration range from 1ng/ml to 3μg/ml. Vitamin K1 (phylloquinone) was added at a concentration of 150ng/ml as an internal standard in each sample. Quantities of MK-5 and MK-6 were estimated using the formulas derived from MK-4 and MK-7 calibration curves, respectively; quantities of MK-8 and MK10 were estimated using the formula derived from MK-9 calibration curve. Values were corrected based on measurement of vitamin K1 internal standards.

### Oxygen Consumption Rate Analysis

Strains were all cultivated under anaerobic conditions overnight in GM17 to obtain biomass for oxygen consumption analysis. For the test under respiration-permissive conditions, 2μg/ml heme was supplemented to obtain cells with functional cytochrome *bd* oxidase. The cells were harvested and washed in PBS once, and OD was standardized to 2 in PBS that had already been saturated with dissolved oxygen (by shaking for 30min at 180rpm in flasks). Bacterial suspension (20ml) was placed in infusion bottles (volume 25ml) with a layer of mineral oil on top to prevent exchange of gas. An oxygen microsensor (Needle-type PSt7-02, PreSens, Germany) was inserted into the bacterial suspension. The reaction was initiated by adding glucose to a concentration of 1% in the bacterial suspension. Dissolved oxygen in the bacterial suspension was followed at room temperature by a Microx 4 oxygen meter (PreSens, Germany) for 20min with an interval of 1min. The absolute value of the slope of the linear correlation between oxygen concentration and time for each measurement was taken as the oxygen consumption rate.

### Primary Metabolite Analysis

Lactate, formate, acetate, acetoin, and ethanol were analyzed by HPLC. The strains were incubated in GM17 media at 30°C overnight under static or aerobic conditions. Cell-free culture supernatant was obtained by pelleting the cells at 17,000×*g* for 5min. To remove proteins, the supernatant was first mixed with 0.5 volume cold Carrez A solution [0.1M K_4_Fe(CN)_6_] and then mixed with 0.5 volume cold Carrez B solution (0.2M ZnSO_4_), and centrifuged at 17,000×*g* for 5min. The supernatant was subjected to HPLC analysis as described by van Mastrigt et al. (2018). Briefly, 25μl sample was injected into HPLC system Ultimate 3,000 (Dionex, Idstein, Germany) equipped with an Aminex HPX-87H column (300×7.8mm). Compounds were eluted by 5mM sulfuric acid at a flow rate of 0.6ml/min at 40°C, identified by UV detectors at 220, 250, and 280nm and quantified by a refractive index detector Shodex RI-101 (Showa Denko K.K., Tokyo, Japan). The quantity of metabolites detected in un-inoculated GM17 medium was used as the initial level to calculate the production/consumption of bacterial metabolites.

### Genome Sequencing

The original strain MG1363 and three selected evolved strains, Evo1, Evo2, and Evo3, were subjected to genome sequencing. For genomic DNA isolation, cells collected from 1ml of an overnight culture (static incubation in GM17) of each strain was used. DNeasy Blood & Tissue Kit (Qiagen, Germany) was used to extract DNA according to the manufacture’s instruction. DNA was sequenced using the Illumina HiSeq Genome Sequencing System (GATC Biotech, Germany), read length 2×150bp. The sequencing reads were deposit under BioProject with accession number PRJNA765529 at National Center for Biotechnology Information (NCBI) Sequence Read Archive (SRA).

Sequenced reads from each genome were mapped to the reference sequence of *L. lactis* ssp. *cremoris* MG1363 (NCBI accession No. AM406671_1) using BWA (Li and Durbin, 2009) with default parameters. In all strains, more than 99.9% of all reads were mapped. The removal of PCR duplicates was carried out using Picard (Picard Toolkit, 2019). In the four sequenced strains, 935–1,141×coverage depths were reached. Variant analysis was performed using GATK’s Haplotype Caller (McKenna et al., 2010; DePristo et al., 2011) to identify single nucleotide polymorphism (SNP) and insertion and deletion (InDel).

### Proteomics Analysis

Strains were cultivated in GM17 media at 30°C overnight (16h) under desired conditions (ST, AE, and RES). Cells were harvested from 1ml cultures, washed and resuspended in 100mM Tris (pH 8), and lysed by a sonication probe twice for 45s. For each strain and condition combination, samples were collected from three independent experiments. For all samples, 40μg proteins were used for sample preparation and analysis. Sample preparation followed the filter-assisted sample preparation protocol (FASP; Wiśniewski et al., 2009). In brief, proteins were reduced with 15mM dithiothreitol, alkylated with 20mM acrylamide, and digested with trypsin. Maximally 5μl prepared sample was injected into a 0.10×250mm ReproSil-Pur 120 C18-AQ 1.9μm beads analytical column (prepared in-house) at a constant pressure of 825bar using a 1-h gradient from 9 to 34% acetonitrile in water with 0.1% formic acid in 50min by a nanoLC-MS/MS (Thermo nLC1000 coupled to a Q Exactive-HFX). MS and MSMS AGC targets were set to 3.106, 50,000, respectively, or maximum ion injection times of 50ms (MS) and 25ms (MSMS) were used. HCD-fragmented (isolation width 1.2m/z, 24% normalized collision energy) MSMS scans of the 25 most abundant 2–5+ charged peaks in the MS scan were recorded in data-dependent mode (threshold 1.2e5, 15s exclusion duration for the selected m/z +/− 10ppm).

The MaxQuant quantitative proteomics software package was used to analyze LCMS data with all MS/MS spectra as described previously (Cox et al., 2014), and the proteome of *L. lactis* MG1363 (UniProt ID UP000000364) was used as the protein database. Perseus was employed for filtering and further bioinformatics and statistical analysis of the MaxQuant ProteinGroups files (Tyanova et al., 2016). Reverse hits were removed; identified protein groups should contain minimally two peptides, of which at least one is unique and one unmodified. The label-free quantitation (LFQ) intensity values were used for *t* test; protein groups differing by a factor of 2 or more (log fold change≤−0.3 or≥0.3) and a *p*-value of 0.05 or less (−log value of *p*≥1.3) were considered significantly different. The quality of nLC-MS/MS system was checked with PTXQC using the MaxQuant result files (Bielow et al., 2016). The protein lists and LFQ intensity values of all samples can be found in Supplementary Data Sheet. The mass spectrometry proteomics data have been deposited to the ProteomeXchange Consortium *via* the PRIDE (Vizcaíno et al., 2016) partner repository with the dataset identifier PXD028721.

### Data Analysis

Where applicable, statistical significance analysis was performed in JASP (0.11.1; Love et al., 2019) using ANOVA unless specified otherwise. *Post hoc* multiple comparisons were conducted using Tukey’s test (two-sided), and in all cases the control group was *L. lactis* MG1363 (^*^p≤0.05).

## Results

### Three Evolved Strains Showed Better Survival and Higher Vitamin K2 Production

**Figure 1:**
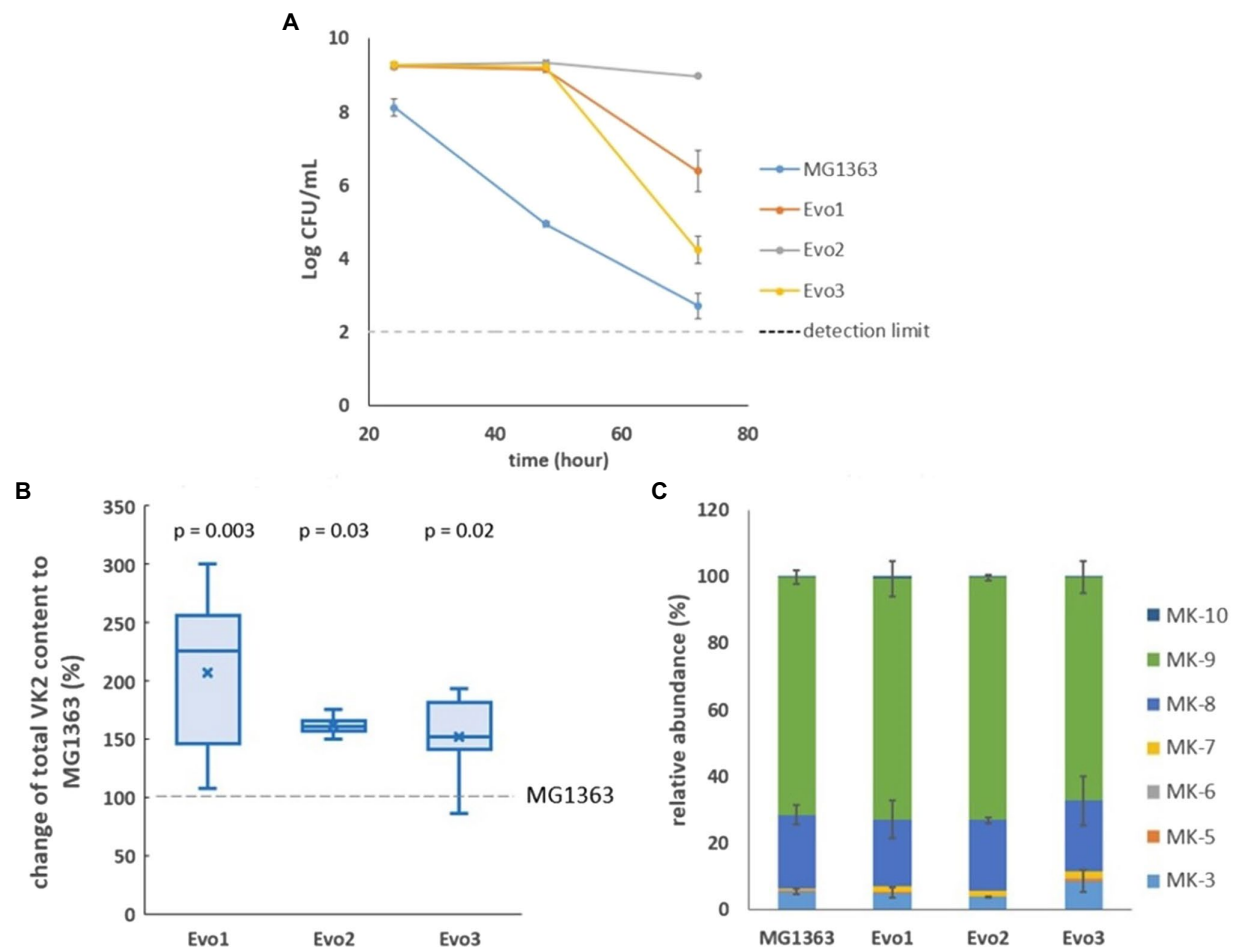
Selection of strains. **(A)** Viable plate count of MG1363 and three evolved strains under aerated conditions after 24-, 48-, and 72-h cultivation in GM17 media at 30°C. Data from biological triplicates, error bars represent SD. **(B)** Differences of vitamin K2 (VK2) content in three evolved strains comparing to strain MG1363, under static fermentation condition for 48h in GM17 media at 30°C. Data from four independent experiments, differences in percentage were calculated from each experiment. Crosses represent mean values in the graph; *p*-values of each strain compared to MG1363 were calculated by paired *t*-tests. **(C)** Relative abundance of different menaquinone forms in strain MG1363 and evolved strains under static fermentation condition for 48h in GM17 media at 30°C. Data from two independent experiments, error bars represent SEM.

After sequential propagation of a culture of *L. lactis* ssp. *cremoris* MG1363 for 100 generations under intensively aerated conditions (shaking at 200rpm in flasks with 10 x headspace of the culture) for 72h in glucose supplemented M17 (GM17) media, we isolated three strains and examined them closely. All three strains (Evo1, Evo2, and Evo3) showed considerably better survival compared to the original strain MG1363 under aerated conditions, as reflected by viable plate count: When all strains were cultivated in GM17 under the aerated conditions employed in the evolution experiment for 72h, the culturability was tested at 24, 48, and 72h (Figure 1A). While the viable plate count of strain MG1363 dropped to 10^5^CFU/ml after 48h in aerated conditions from the initial 10^9^CFU/ml (not shown), the three evolved strains all maintained at 10^9^CFU/ml. After 72-h aerated cultivation, viable plate count of strain MG1363 further dropped to 10^2^–10^3^CFU/ml, while strains Evo1, Evo2, and Evo3 were still at 10^6^, 10^9^, and 10^4^CFU/ml, respectively.

We also examined the vitamin K2 content in three evolved strains in comparison to the original strain under static fermentation conditions, as this is the most relevant condition for applications. The total vitamin K2 content in MG1363 was 80±19nmol/g cell dry weight. The three evolved strains showed 50–110% higher average total vitamin K2 content than the original strain (Figure 1B). The relative abundance of MK forms produced by evolved strains are similar to MG1363: 65–70% MK-9, 20% MK-8, 5–9% MK-3, and minor amount of other forms including MK-10, MK-5 to MK-7 (Figure 1C).

### Growth and Survival of Evolved Strains in Various Conditions

**Figure 2:**
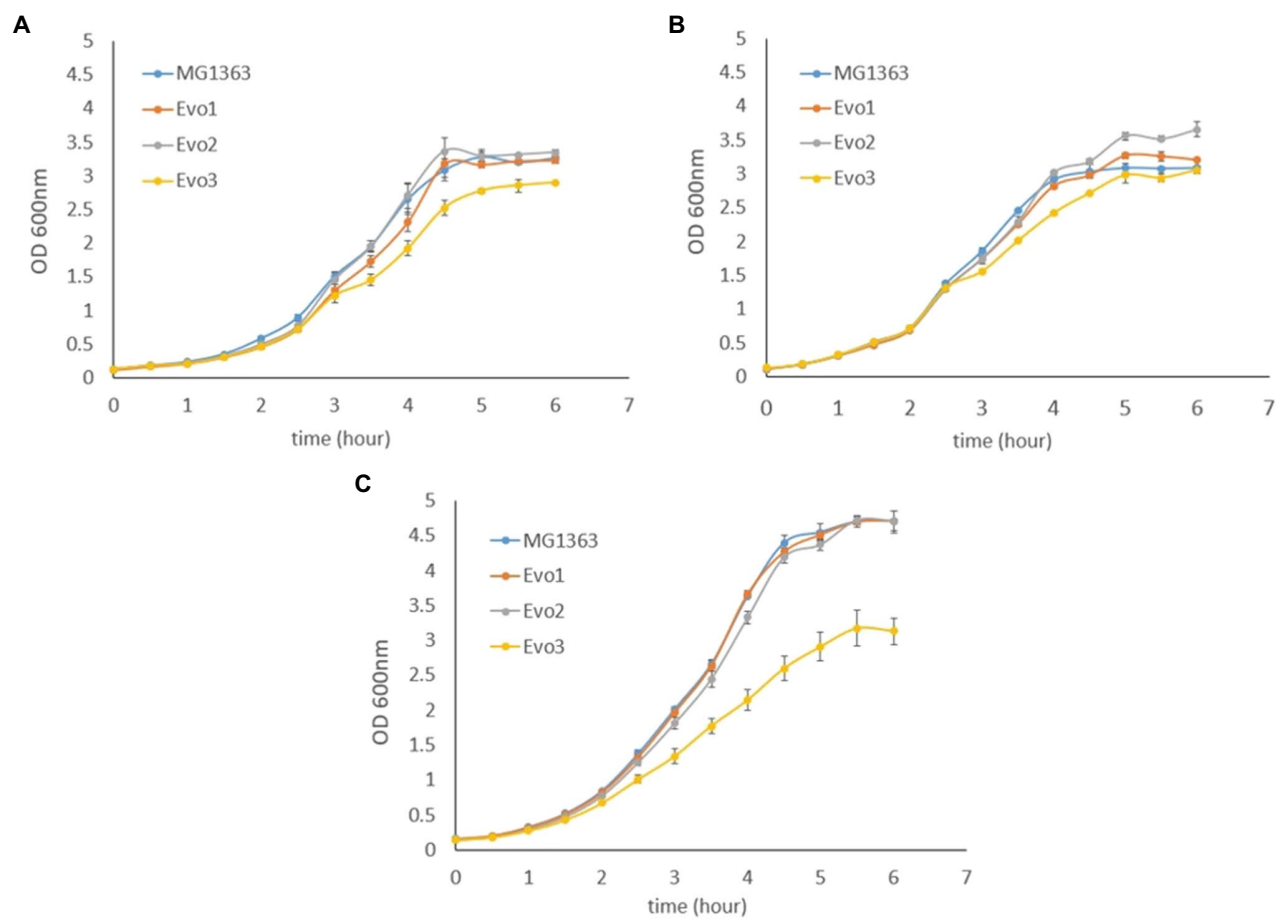
Growth curves based on optical density (OD) of MG1363 and evolved strains under **(A)** static fermentation, **(B)** aerated, and **(C)** respiration-permissive conditions in GM17 media at 30°C. Data from biological triplicates, error bars represent SD.

As the evolved strains showed favorable traits mentioned above, we further examined the growth and survival of evolved strains under various conditions, namely static, aerated, and respiration-permissive conditions in GM17 media (Figure 2). Under static and aerated conditions, the evolved strains behaved similar to MG1363: They all reached stationary phase (around OD 3.0–3.5) in 5h, and Evo3 showed slightly lower turbidity (about 0.5 lower in OD) than the other strains when reaching stationary phase under static cultivation. Under respiration-permissive condition, MG1363, Evo1, and Evo2 all reached OD values of 4.5–5.0 in 5h, while Evo3 only reached OD of 3.0 at stationary phase.

**Figure 3:**
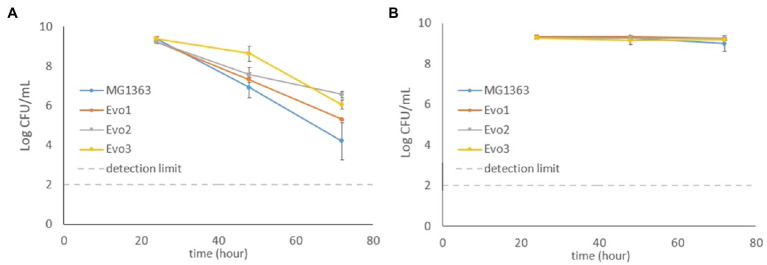
Viable plate counts of MG1363 and evolved strains throughout 72h under **(A)** static fermentation and **(B)** respiration-permissive conditions in GM17media at 30°C. Data from biological triplicates, error bars represent SD.

Besides survival under aerated conditions that was used for the selection of evolved strains (Figure 1A), the survival (reflected by viable plate counts) of evolved strains and MG1363 was also tested under static and respiration-permissive conditions for 72h (Figure 3).

Under static condition, the viable counts of MG1363 dropped to 10^7^CFU/ml at 48h from the initial 10^9^CFU/ml and further decreased to 10^4^CFU/ml at 72h. All three evolved strains showed similar decrease along time, but were constantly 0.5–2 log CFU/ml higher than MG1363 at 48 and 72h (*p*<0.05 for Evo2 and Evo3). Under respiration-permissive condition, all strains maintained high viable counts at 10^9^CFU/ml throughout the 72h.

### Oxygen Consumption Rate in Evolved Strains

**Figure 4:**
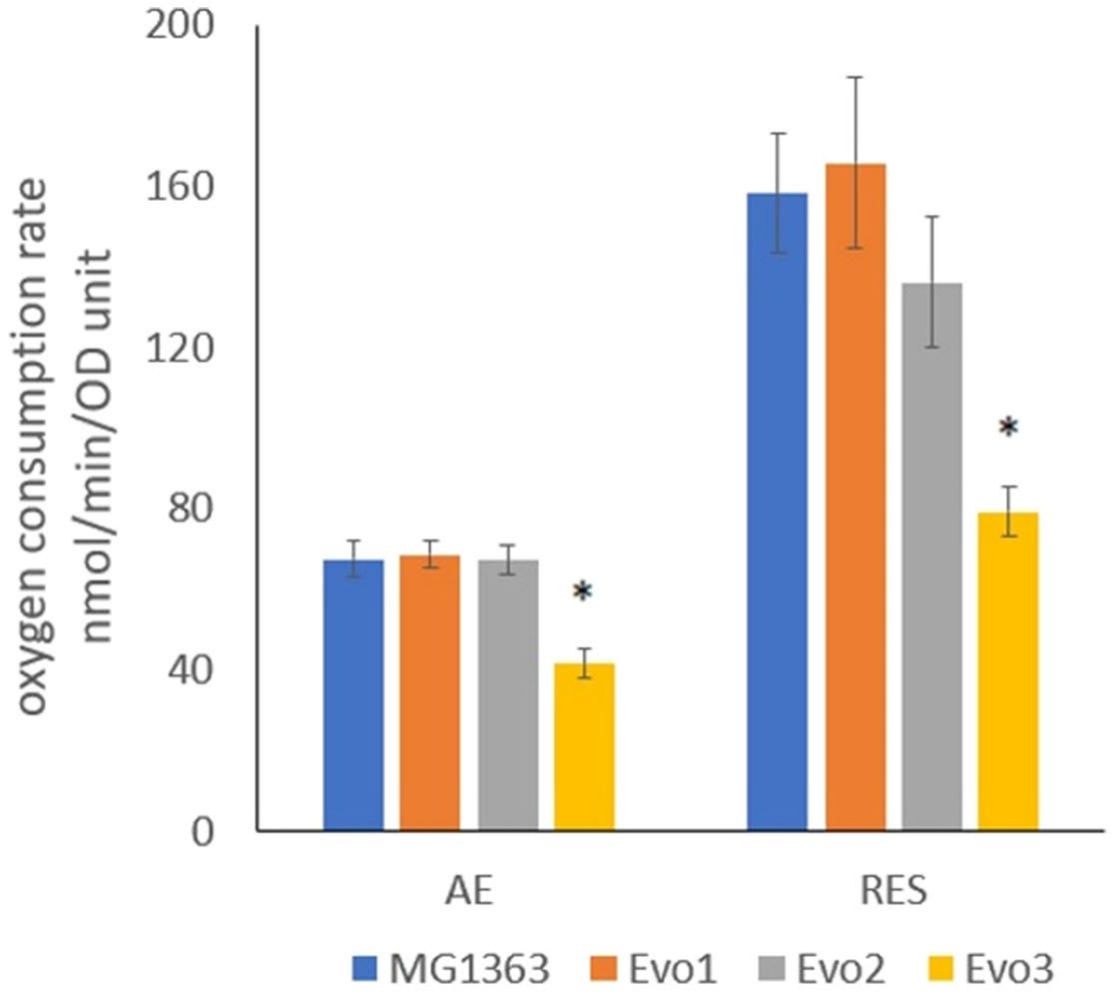
Oxygen consumption rate of strain MG1363 and evolved strains under aerobic and respiration-permissive conditions. Cells for this test were obtained from overnight cultures in GM17 media at 30°C under anaerobic condition, and in addition 2μg/ml heme was supplemented to the culture for obtaining cells used for respiration-permissive (RES) condition oxygen consumption test. PBS-washed cells were suspended in air-saturated PBS (OD standardized all to 2) for oxygen consumption test at room temperature. Reaction was initiated by adding 1% glucose. Data from four independent experiments, error bars represent SEM. *Indicates significant difference compared to MG1363 under the same condition (*p*≤0.05).

As the evolved strains were obtained from aerated conditions, oxygen consumption by each strain was a relevant phenotype. The oxygen consumption rates in evolved strains and MG1363 were examined under aerobic and respiration-permissive conditions (Figure 4). The biomass was obtained by cultivating each strain in GM17 media overnight anaerobically (heme added to the biomass for respiration-permissive condition), washed and resuspended in air-saturated PBS for the oxygen consumption test. Under aerobic condition, MG1363, Evo1, and Evo2 showed similar oxygen consumption rate of about 70nmol/min/OD unit, while Evo3 showed a significantly lower rate of 40nmol/min/OD unit. Under respiration-permissive condition, the oxygen consumption rates of all strains were doubled compared to the values found under aerobic condition: MG1363, Evo1, and Evo2 reached 140–160nmol/min/OD unit, while Evo3 reached 80nmol/min/OD unit.

### Evolved Strains Showed High Resistance to H_2_O_2_

**Figure 5:**
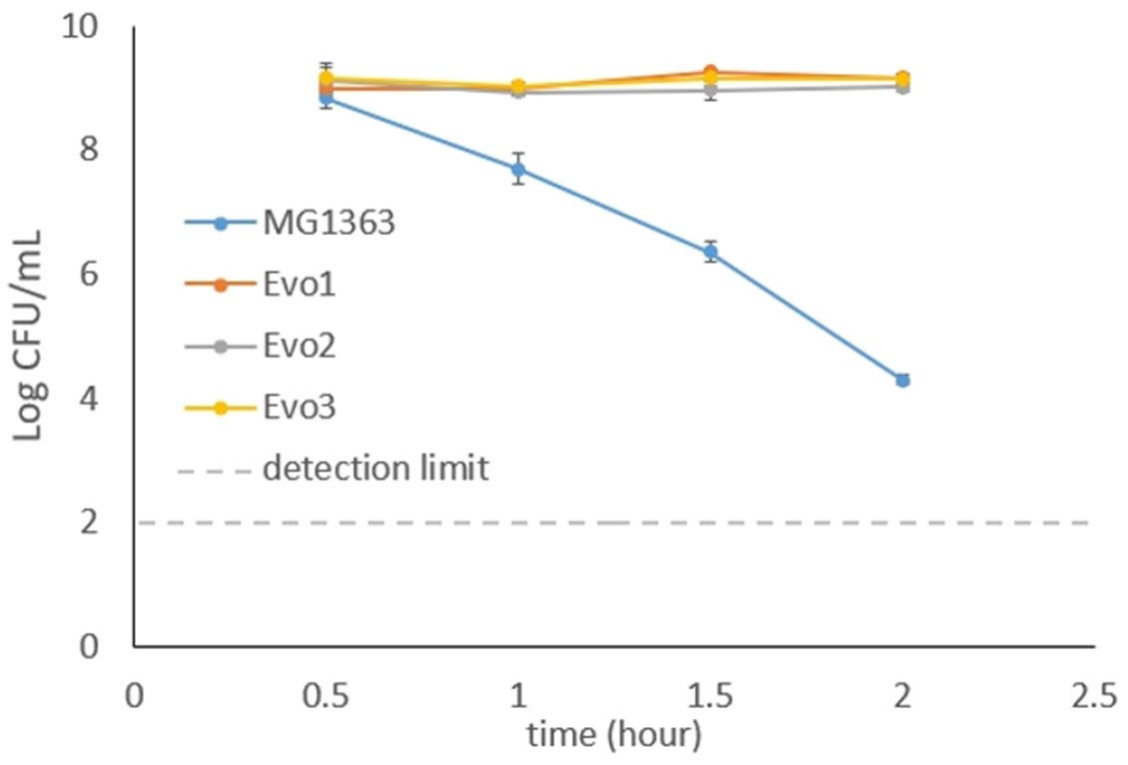
Viable plate counts of strain MG1363 and evolved strains after exposure to 5mM H_2_O_2_. Cells in the early exponential phase were treated with H_2_O_2_ in GM17 media, incubated statically at 30°C. Data from biological triplicates, error bars represent SD.

In addition to the previous observation that the evolved strains maintained high survival under aerated cultivation conditions, we also tested the strains for resistance to oxidative stress caused by H_2_O_2_. Cells from early log phase (OD_600_=0.2) from each strain were exposed to 5mM H_2_O_2_ in GM17 media, and the viable plate count was monitored for 2h (Figure 5). The viable plate count of MG1363 kept decreasing after 0.5-h exposure to H_2_O_2_, and dropped to 10^4^CFU/ml after 2h. In contrast, all evolved strains maintained viable counts of 10^9^CFU/ml throughout the 2-h exposure to H_2_O_2_.

### Acidification Capacity of Evolved Strains

**Figure 6:**
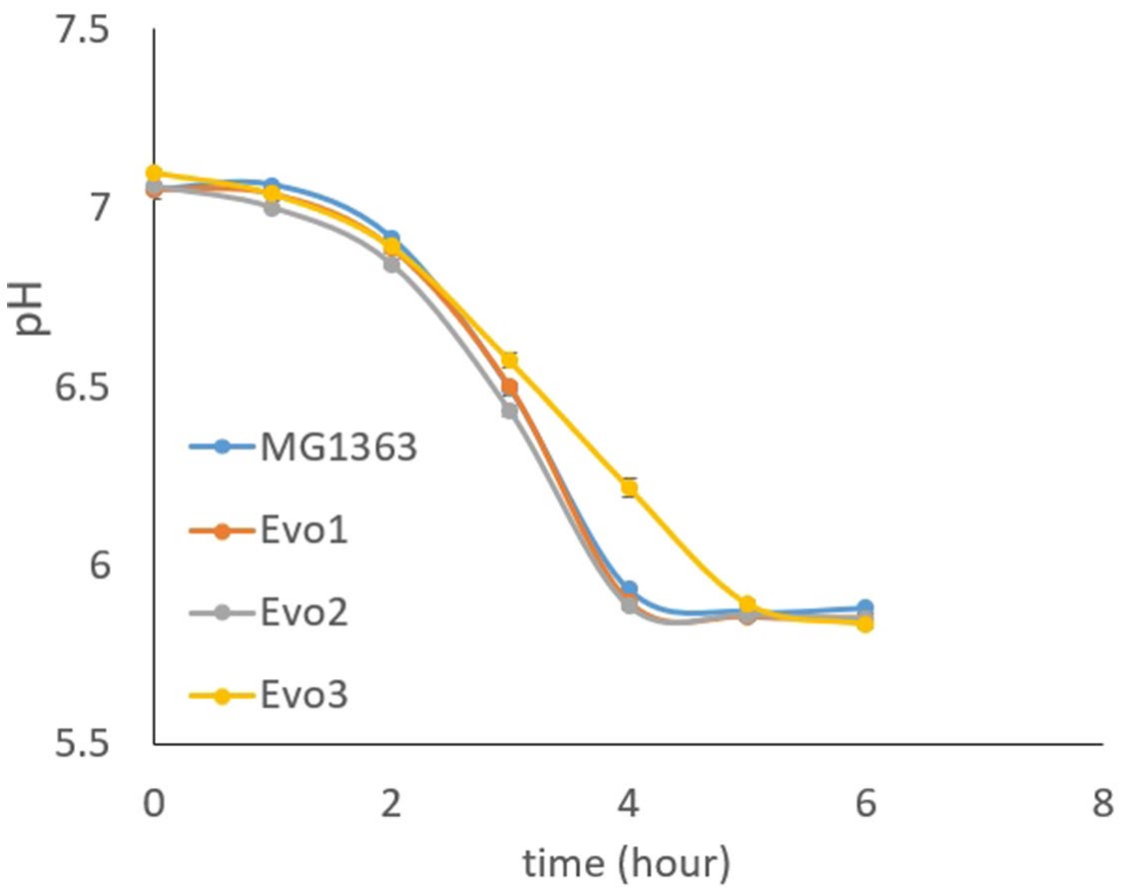
Acidification capacity of MG1363 and evolved strains. Test started with cells at OD=0.1 in GM17 media, incubated statically at 30°C.

As the acidification capacity is an important feature for lactic acid bacteria as starter cultures, we also examined the evolved strains for their ability to lower the pH in GM17 media. Cells from each strain were inoculated at OD of 0.1 in GM17 media and the pH was followed for 6h. MG1363, Evo1, and Evo2 lowered the pH from 7 to below 6 in 4h, while Evo3 showed 1-h delay than the other strains in reducing the pH to lower than 6 (Figure 6).

### Evo1 and Evo2 Produced Less Lactate and More Acetate Under Aerobic Condition

**Figure 7:**
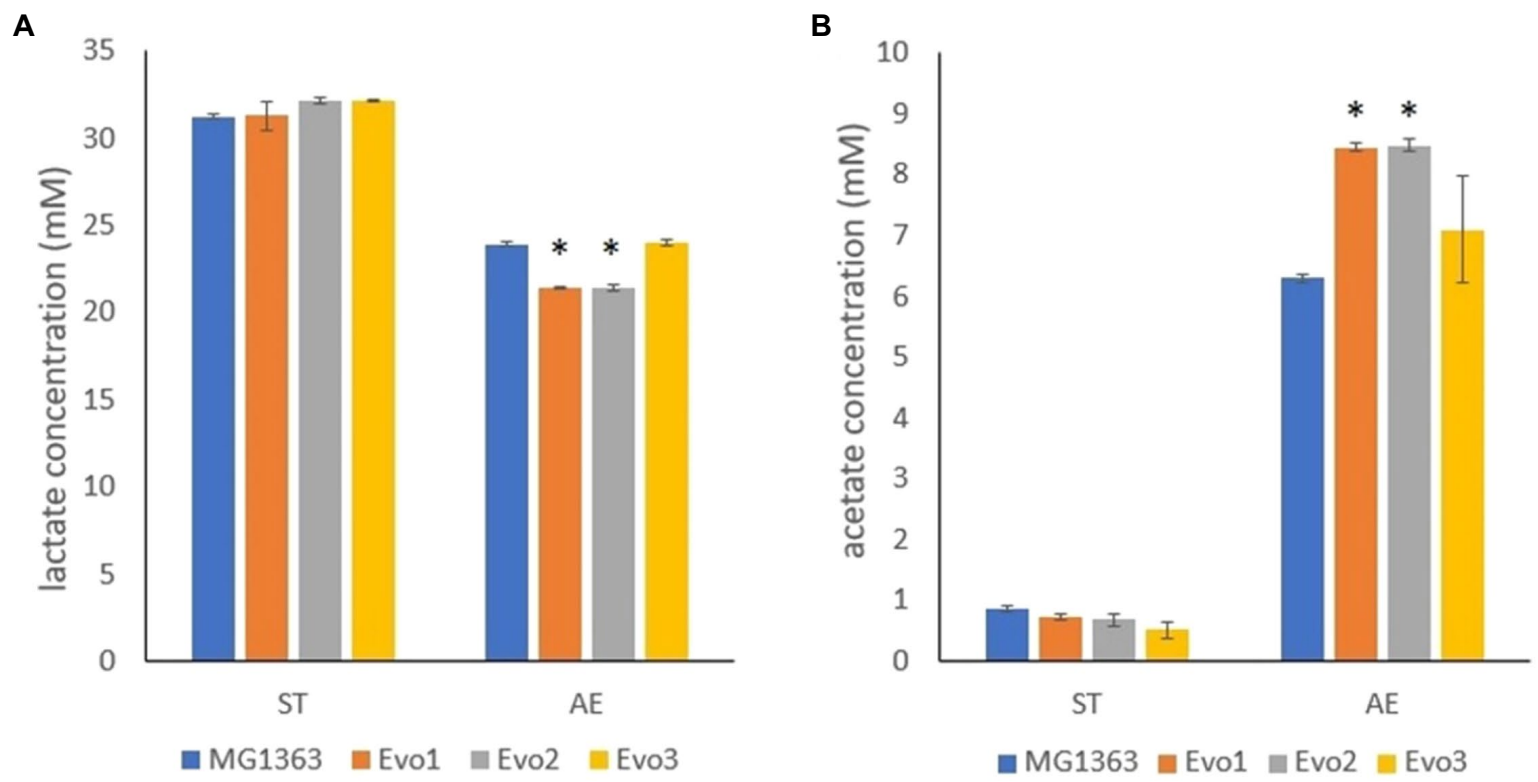
Primary metabolites produced by MG1363 and evolved strains. **(A)** Lactate and **(B)** acetate concentration from the supernatant of cultures in GM17 at 30°C under indicated conditions. Data from three independent experiments, error bars represent SEM. *Indicates significant difference compared to MG1363 under the same condition (*p*≤0.05).

The primary metabolites were examined in the overnight cultures of original and evolved strains under static and aerobic condition in GM17 media. Among all tested metabolites, namely lactate, acetate, formate, acetoin, and ethanol, significant differences between strain MG1363 and evolved strains were observed for lactate and acetate under aerobic condition (Figure 7). Under anaerobic condition, all strains produced about 32mM lactate and 0.5–1mM acetate. Under aerobic condition, MG1363 produced 25mM lactate and 6.5mM acetate, Evo3 produced the same amount of lactate as MG1363 and about 1mM more acetate, while Evo1 and Evo2 produced 2.5mM less lactate but 2mM more acetate than MG1363.

### Evolved Strains Showed Common Mutations in *ldh* and *gapB* Gene

Whole genome sequencing revealed the mutations in evolved strains comparing to strain MG1363 (Table 1). All three evolved strains contained SNPs in genes *ldh* (*llmg_1120*), *ps435* (*llmg_2107*), and *gapB* (*llmg_2539*) encoding proteins L-lactate dehydrogenase, hypothetical protein, and glyceraldehyde 3-phosphate dehydrogenase, respectively. Evo1 and Evo2 shared a SNP in llmg_0907 encoding an YlbN-like protein. Evo3 contained two unique mutations: a SNP in *ftsL* (*llmg_1680*) encoding a cell division protein and a deletion in *purR* (*llmg_2551*) encoding a *pur* operon repressor. A few more unique mutations in Evo2 and Evo3 were revealed, but these were in pseudogenes or noncoding sequences; except for a SNP in Evo2 that could be in the promoter region of *rplA* (*llmg_2276*), the rest were not expected to have regulatory effects on gene expression. No mutations were identified in genes that are predicted to be involved in vitamin K2 biosynthesis in any of the evolved strains.

**Table 1:** Mutations in evolved strains compared to MG1363.

Strains		Position	Variation	Sequence change	Gene	Protein and ID	Function	Amino acid change
Unique mutations	Evo2	670,701	INS	G -> GGA	*llmg_pseudo_18*	Pseudo	Transposase and inactivated derivatives	Non-coding
	Evo2	2,233,340	SNP	T -> A	20bp downstream of *llmg_2275*, 43bp upstream of *rplA* (*llmg_2276*)	Downstream of: putative membrane protein (A2RNF2) Upstream of: 50S ribosomal protein L1 (A2RNF3)	Putative membrane protein/Ribosomal protein L1	Non-coding
	Evo3	612,538	SNP	G -> T	295bp up stream of *llmg_pseudo_14*, 19bp downstream of *llmg_0623*	Downstream of: abortive phage resistance protein abiP (A2RIX6)	Abortive phage resistance protein	Non-coding
	Evo3	1,655,780	SNP	G -> A	*ftsL* (*llmg_1680*)	Cell division protein (A2RLT3)	Protein required for the initiation of cell division	L69F
	Evo3	2,510,291	DEL	AT -> A	*purR* (*llmg_2551*)	Pur operon repressor (P0A400)	Adenine/guanine phosphoribosyltransferases and related PRPP-binding proteins	Frameshift (last 13 amino acids)
Common mutations	Evo1, Evo2	873,610	SNP	C -> A	*llmg_0907*	Hypothetical protein, YlbN-like protein (A2RJP8)	Predicted metal-binding, possibly nucleic acid-binding protein	T9K
	Evo1, Evo2,	1,081,552	SNP	T -> A	*ldh* (*llmg_1120*)	L-lactate dehydrogenase (A2RKA4)	Malate/lactate dehydrogenases	V212D
	Evo3	1,081,502		A -> T				K195N
	Evo1, Evo2, Evo3	2,090,425	SNP	C -> G	*ps435* (*llmg_2107*)	Hypothetical protein (A2RMY7)		K13N
	Evo1, Evo2, Evo3	2,492,912	SNP	G -> A	*gapB* (*llmg_2539*)	Glyceraldehyde 3-phosphate dehydrogenase (A2RP55)	Glyceraldehyde-3-phosphate dehydrogenase/erythrose-4-phosphate dehydrogenase	A203V

### Proteomes of Evolved Strains Differed Most From MG1363 Under Aerobic Condition

The proteomes of evolved strains and strain MG1363 were examined when cells were cultivated under static, aerobic and respiration-permissive conditions in GM17 media overnight. In total 1,387 proteins were quantified by the proteomics analysis, out of the 2,383 proteins predicted for MG1363 proteome (Uniprot proteome ID UP000000364).

Among the three tested cultivation conditions, all three evolved strains showed the highest numbers of proteins with significantly different production level (cut-off *p*≤0.05, fold change≥2) compared to MG1363 under aerobic conditions (Table 2). Evo1 and Evo2 had 17 and 16 proteins differently produced compared to MG1363 under the aerobic condition, while under static and respiration-permissive condition only 2–3 proteins were identified to be differently produced. Evo3 showed the biggest proteome change compared to MG1363, and the numbers of differentially produced proteins under static, aerobic, and respiration-permissive conditions were 234, 300, and 292, respectively. Under all tested conditions, a significant number of the differentially produced proteins in Evo3 compared to MG1363 are identified to be ribosomal proteins and proteins involved in purine nucleotide and amino acids biosynthesis (Supplementary Figures S1, S2).

**Table 2:** Numbers of differentially produced proteins in the evolved strains compared to MG1363 under different cultivation conditions.

No. of proteins	Conditions	ST	AE	RES
Evo1	Overproduction	0	5	1
	Underproduction	2	12	1
	Total different	2	17	2
Evo2	Overproduction	2	6	1
	Underproduction	1	10	2
	Total different	3	16	3
Evo3	Overproduction	126	172	160
	Underproduction	108	128	132
	Total different	234	300	292

**Figure 8:**
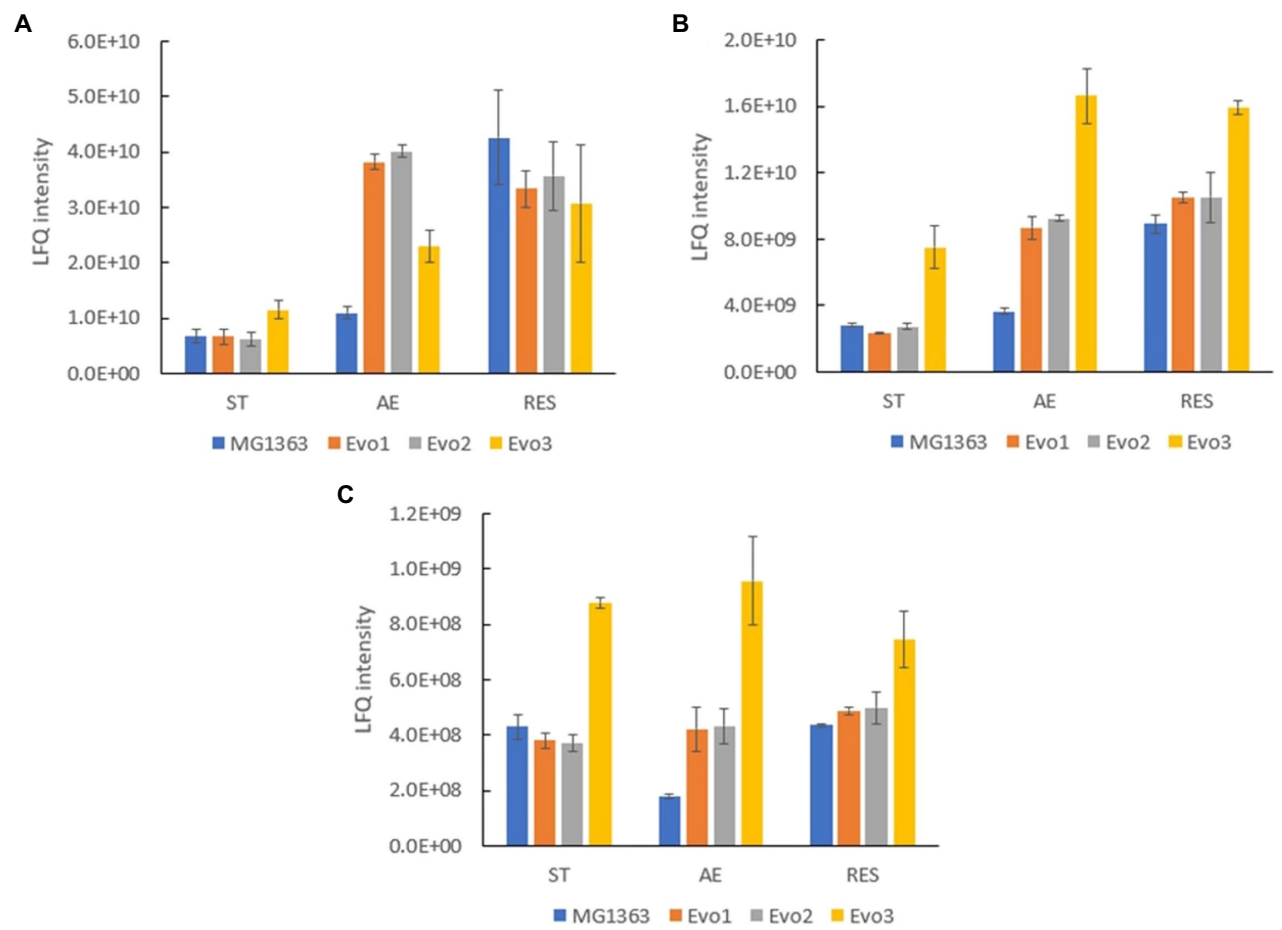
Quantity of common differentially produced proteins in evolved strains than MG1363 under different cultivation conditions. Label-free quantitation (LFQ) intensities of **(A)** glyceraldehyde 3-phosphate dehydrogenase, GapA (A2RIN9), **(B)** universal stress protein A2, UspA2 (A2RK64), and **(C)** formamidopyrimidine-DNA glycosylase, MutM (A2RI84). Samples were from three independent experiments, error bars represent SEM.

Under the aerobic condition, three proteins were identified to be significantly (*p*≤0.05, fold change≥2) overproduced in all evolved strains compared to MG1363 (Supplementary Figure S3): glyceraldehyde 3-phosphate dehydrogenase (A2RIN9), universal stress protein A2 (A2RK64) and formamidopyrimidine-DNA glycosylase (A2RI84; Table 3). Quantities of these three proteins in all strains under different cultivation conditions were examined closely (Figure 8). Under static condition, the levels of the three proteins in Evo1 and Evo2 were similar to that in MG1363, while Evo3 showed doubled amount compared to the rest. Under aerated conditions, the levels of the three proteins in the evolved strains were in general 2–4 times higher than in MG1363. Under respiration-permissive condition, the amounts of the three proteins in MG1363 were close to the evolved stains, about 2–4 times higher than that of MG1363 under aerobic conditions.

**Table 3:** Common proteins overproduced in all mutants compared to MG1363 under aerated conditions.

Protein ID	Protein name/Gene name (GN)	GO terms and functions
A2RIN9	Glyceraldehyde 3-phosphate dehydrogenase GN=gapA	Oxidoreductase activity, acting on the aldehyde or oxo group of donors, NAD or NADP as acceptor. Glucose metabolic process.
A2RK64	Universal stress protein A2 GN=uspA2	
A2RI84	Formamidopyrimidine-DNA glycosylase GN=mutM	Involved in base excision repair of DNA damaged by oxidation or by mutagenic agents. Acts as DNA glycosylase that recognizes and removes damaged bases.

Proteomics analysis did not reveal many cases of differential production of proteins encoded by genes with mutations in evolved strains compared to MG1363 in all cultivation conditions (Supplementary Table S1), with the exception that *purR* encoded protein was at least 10 times less in Evo3 than the other strains in all conditions. Moreover, we also did not observe significant changes in proteins that are predicted to be involved in the vitamin K2 biosynthesis pathway among all strains in the three tested cultivation conditions: static, aerobic, and respiration-permissive condition (Supplementary Table S2).

## Discussion

Enhancing vitamin K2 content in lactic acid bacteria represented by *L. lactis via* non-GM approaches is highly relevant for enrichment of this valuable vitamin in fermented foods and supplements. Since in previous studies, it was observed that aerated cultivation conditions increased vitamin K2 content in *L. lactis* (Liu et al., 2019), and because vitamin K2 has been suggested to contribute to oxidative stress resistance in bacteria (Søballe and Poole, 2000; Vido et al., 2005), we performed laboratory evolution through aerated cultivation using the model strain *L. lactis* MG1363. Prolonged cultivation time (72h) in aerobic conditions was applied for each passage during the evolution experiment, to impose strong selection pressure as evidenced by the decline in culturable cells of the original wild-type cells along this time line.

The evolved strains did not only show significantly higher stationary phase survival under aerated conditions, but also showed 50–110% higher total vitamin K2 content than the original strain under static fermentation condition, which is the most relevant cultivation condition for (food) fermentations with *L. lactis*. This increase is considerable, comparing to the 30–100% increase obtained by selecting resistant mutant against a menaquinone analogue for *Bacillus subtilis* (Sato et al., 2001; Tsukamoto et al., 2001). Liu et al. (2019) reported that aerated cultivation conditions and a different carbon source such as fructose, improved vitamin K2 content in MG1363 compared to static cultivation with glucose. Here, we could reproduce this result in strain MG1363, but not with the evolved variants of this strains (Supplementary Figure S4). Aeration and different carbon source did not result in the same level of increase of vitamin K2 content in the evolved strains as they did for MG1363, and in some cases even lowered the vitamin K2 content in the evolved strains.

As the evolved strains showed desirable traits in vitamin K2 content, essential aspects for application of these strains, such as growth performance, acidification capacity, and oxidative stress resistance, were studied. Evo1 and Evo2 performed as good as MG1363 in growth and acidification in static fermentation, while Evo3 showed a slightly lower turbidity and a delay in acidifying the culture medium. This could be explained by the unique mutation in Evo3 in the cell division protein encoding gene *ftsL* and the *pur* operon repressor *purR*, the latter supported also by proteomics analysis, showing a big global proteome change in ribosomal proteins, purine and amino acid synthesis proteins in Evo3 compared to the other strains. The PurR regulon has indeed been shown to include promoters in nucleotide metabolism, (p)ppGpp metabolism, translation-related functions and more (Jendresen et al., 2012), and mutations in *purR* can thus influence the cell growth, metabolism, and stress response. The mutation in *ftsL* can result in hindered cell growth as well as morphological changes (Guzman et al., 1992), and both factors may contribute to the slightly lower turbidity of Evo3 comparing to other strains under static fermentation. Evo3 also differed from the other strains with respect to growth under respiration-permissive condition. MG1363, Evo1, and Evo2 all showed nearly doubled biomass in respiration-permissive conditions as compared to static and aerated cultivation, which is a well-known effect of aerobic respiration in *L. lactis*. Evo3 did not show increased biomass under respiration-permissive condition. An explanation for this observation can be derived from the proteome data (Supplementary Table S3): Evo3 showed a 4–6 times lower amount in NADH dehydrogenase NoxA and slightly less NoxB than the other strains under all tested conditions. NoxA and NoxB, are membrane bound type II NADH dehydrogenases in *L. lactis* which, together with menaquinone and cytochrome bd forms functional ETC (Brooijmans et al., 2007; Tachon et al., 2010). The reduced amount of NoxA and NoxB in Evo3 could have compromised the efficiency of the ETC under respiration-permissive condition. Moreover, Evo3 also produced about 50% less NoxE (Supplementary Table S3), another NADH dehydrogenase that directly donates electrons from NADH to oxygen. Together with the reduced amount in NoxA and NoxB, this could explain the lower oxygen consumption rate in Evo3 under aerobic and respiration-permissive conditions compared to the other strains.

Given the conditions used during the laboratory evolution process, the evolved mutants were selected for both resistance to acid and starvation stress in stationary phase, as well as to oxidative stress. For the improved stationary phase survival, it is likely that the three evolved strains adopted different approaches: Evo1 and Evo2 produced less lactate in aerated conditions (Figure 7), which could be beneficial for reducing acid stress for the two strains. Evo3 showed unique proteome profile, where a whole group of proteins involved in arginine biosynthesis/metabolism were differentially produced comparing to the other strains (Supplementary Figure S2). *Lactococcus lactis* is known to utilize arginine as an alternative energy source when the sugar source is deprived (Brandsma et al., 2012), and the unique profile of arginine biosynthesis/metabolism proteins of Evo3 could offer advantage to this strain when facing starvation stress in the stationary phase. This was also in line with the observation that Evo3 was one of the best survivors throughout prolonged cultivation in static fermentation conditions (Figure 3A).

The high resistance of evolved strains against oxidative stresses was also confirmed separately by exposing bacteria to hydrogen peroxide, and putative factors involved were identified in our proteomics analysis. In the proteomics analysis, the three proteins that were found to be overproduced in each of the evolved strains under aerated conditions were identified (Supplementary Figure S3). These three proteins, glyceraldehyde 3-phosphate dehydrogenase (GapA), universal stress protein A2 (UspA2), and formamidopyrimidine-DNA glycosylase (MutM) could explain the oxidative stress-resistant phenotype of the evolved strains. Solem et al. (2003) reported that gapA is only expressed under certain stress conditions, and Rochat et al. (2012) reported that overproduction of GapA led to increased resistance to H_2_O_2_ in MG1363. In this study, proteomics analysis revealed that in MG1363, GapA production was low in static condition, increased 2-fold in aerated conditions and 8-fold in respiration-permissive condition, where in evolved strains GapA was produced at very high level in both aerated and respiration-permissive conditions. Similar trends were also observed for UspA2 and MutM, proteins involved in universal stress response and DNA repair upon oxidative damages, respectively. In the evolved strains, these proteins were produced at high levels in both the aerated conditions and respiration-permissive conditions, but in MG1363 they were only present at high levels under respiration. A high degree of resistance against stresses is one of the known traits for *L. lactis* when achieving functional respiration (Duwat et al., 2001; Rezaïki et al., 2004); this was confirmed for MG1363 in this study, and also supports the explanation on the oxidative stress-resistant phenotype of the evolved strains: Key proteins represented by GapA, UspA2, and MutM were overproduced in these strains upon oxidative stress, to a similar level as in cells undergoing respiration, and that most likely offered protection to cells.

Common mutations in genes *ldh* and *gapB* were identified in the evolved strains, but the encoded proteins produced in evolved strains were at a similar level as in MG1363 (Supplementary Table S4). However, the protein quantity does not always reflect possible changes in the activity and functionality caused by the mutations, and further studies are required to obtain a more detailed characterization of relevant enzyme activities. It is conceivable that the difference in lactate and acetate production in Evo1 and Evo2 compared to the wild type under aerobic condition could be a result of the SNP in the lactate dehydrogenase encoding gene, as the quantity of other proteins involved in pyruvate metabolism could not explain the difference in lactate and acetate production otherwise (Supplementary Table S4). Moreover, the mutation in GapB could influence the level of GapA, of which a higher protein quantity was observed in the evolved strains under the aerated conditions. It has been reported that *gapA* and *gapB* both encode glyceraldehyde 3-phosphate dehydrogenases (GAPDH), and that GapB mainly performs GAPDH activity in *L. lactis* during normal growth, while GapA activity increases under stressed conditions (Willemoës et al., 2002).

Although most phenotypes of the evolved strains, especially the high resistance against oxidative stresses, could be explained by genomics or proteomics data, we could not fully elucidate the mechanism explaining increased vitamin K2 content in the evolved strains with the same collection of data: The evolved strains did not show any difference in gene sequence or protein production in the vitamin K2 synthesis pathway compared to strain MG1363 (Supplementary Table S2). However, proteomics data obtained in this study were from stationary phase cells to avoid variations caused by the timing of protein production and different growth rates of the strains and thus do not reflect the dynamics during cell growth. Gene expression data in exponential phase cells could provide extra insight into any possible difference in regulation of vitamin K2 production in evolved strains. Moreover, vitamin K2 is a group of secondary metabolites. Its production is not only determined by expression level or activity of genes and proteins directly involved in vitamin K2-specific biosynthesis pathway, but also by fluxes toward the precursors or competing reactions. Therefore, the increased vitamin K2 content in evolved strains can be best understood by using a genome-scale metabolic model in future studies.

Despite a clear correlation, the high resistance against oxidative stresses in the evolved strains could not be linked directly to the elevated vitamin K2 content in *L. lactis*. Given the challenges of exogenously supplementing the hydrophobic long-chain MKs, opportunities could be provided by supplementing additional short-chain MKs to *L. lactis*, as previously reported by Rezaïki et al. (2008), who showed that exchange/exogenous supplementation of short-chain MKs in bacteria can activate respiration and stimulate growth in group B streptococcus. Supplemented short-chain MKs can possibly be converted to the native long-chain MK forms in bacteria including *L. lactis*, by a widely conserved isoprenyl diphosphate synthase (part of MK biosynthesis pathway, homologs of IspB described in *Escherichia coli*; Wang and Ohnuma, 2000; Franza et al., 2016; Bøe and Holo, 2020), allowing investigation on the effect of elevated vitamin K2 content in *L. lactis*. Nevertheless, to confirm the physiological roles of vitamin K2/MKs in *L. lactis*, studies can be best performed using dedicated *L. lactis* mutants with different MK profiles but otherwise identical genetical background.

Given the wide applications in fermented food products, *L. lactis* provides unique opportunities for vitamin K2 fortification in our diets. This holds particularly true for the long-chain vitamin K2 forms, which shows a longer half-life in the human body allowing contributions to additional health benefits in vascular and bone health associated with vitamin K2 intake (Gast et al., 2009; Beulens et al., 2013; Schwalfenberg, 2017; Zwakenberg et al., 2017). Although the delivery of these hydrophobic molecules by bacteria cells to the human body remains a challenge and also deserves future attention, efforts to increase the content of vitamin K2 in food grade producers like *L. lactis* is a valuable first step to take.

The evolved *L. lactis* strains obtained and their reported phenotypes highlight the potential of laboratory evolution as a non-GM approach to obtain dairy starters with desired functions in industrial applications. Firstly, the evolved strains, especially Evo1 and Evo2, retained essential features of the original strain in terms of growth and acidification capacity; secondly, the increased vitamin K2 content in the evolved strains enables better fortification of this valuable vitamin in fermented products; finally, the high resistance to oxidative stresses in the evolved strains is desirable for optimal performance of starter cultures (Ghandi et al., 2012; Cretenet et al., 2014; Dijkstra et al., 2014), as *L. lactis* is often subjected to oxidative stresses associated with preservation procedures like spray drying, or linked to processing operations such as stirring in the first stages of cheese manufacturing.

## Conclusion

In this study, we obtained three strains evolved from *L. lactis* ssp. *cremoris* MG1363 by sequential aerated cultivation. Most evolved strains retained essential features of the original strain in terms of growth and acidification performance. In addition, the evolved strains showed not only increased vitamin K2 content but also high resistance against oxidative stresses comparing to the original strain. Genome and proteome analysis provided explanations for most of the phenotypes observed for evolved strains. The laboratory evolution approach therefore showed great potential in obtaining non-GM dairy starters in fermentation industry, where traits of starter cultures such as resistance to oxidative stress, and potentials for enrichment of valuable vitamins like K2, are desired. In conclusion, this study demonstrated a non-GM approach to obtain vitamin K2 overproducers that are highly relevant for food applications, and contribute to the understanding of oxidative stress resistance in *L. lactis*.

## Data Availability Statement

The datasets presented in this study can be found in online repositories. The names of the repository/repositories and accession number(s) can be found below: ProteomeXchange (PXD028721) and NCBI (PRJNA765529).

## Author Contributions

YL, TA, and ES conceived the study and wrote the manuscript. YL designed and executed the experiments and carried out the data analysis and interpretation. AG contributed to the phenotypical characterization of evolved strains in growth, survival, and acidification. SB obtained the proteomics data. All authors contributed to the article and approved the submitted version.

## Funding

The work was subsidized by the Netherlands Organization for Scientific Research (NWO) through the Graduate Program on Food Structure, Digestion, and Health.

## Conflict of Interest

The authors declare that the research was conducted in the absence of any commercial or financial relationships that could be construed as a potential conflict of interest.

## Publisher’s Note

All claims expressed in this article are solely those of the authors and do not necessarily represent those of their affiliated organizations, or those of the publisher, the editors and the reviewers. Any product that may be evaluated in this article, or claim that may be made by its manufacturer, is not guaranteed or endorsed by the publisher.
