# Supplementary material for: Lactococcus lactis Mutants Obtained From Laboratory Evolution Showed Elevated Vitamin K2 Content and Enhanced Resistance to Oxidative Stress
Source: Front Microbiol. 2021 Oct 14;12:746770. doi: 10.3389/fmicb.2021.746770 (PMC8551700; doi:10.3389/fmicb.2021.746770)
Supplement: Supplementary file 1 [file Image_1.pdf]

Supplementary materials – Figures

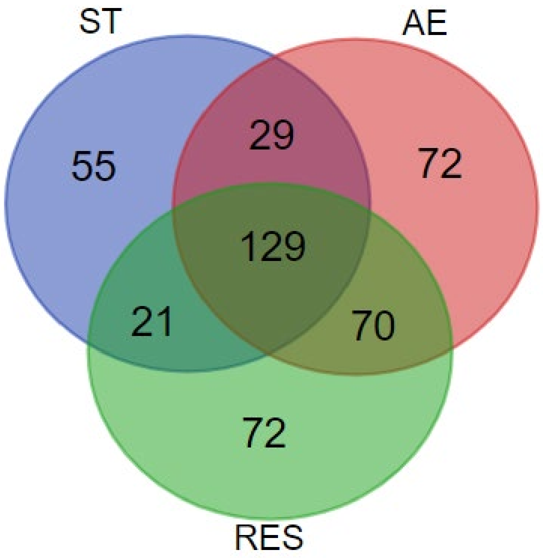

Figure S1. Venn graph showing the differentially produced proteins in Evo3 versus MG1363 in all three tested conditions. There are 129 proteins showing differential production between the two stains in all three conditions. ST, static; AE, aerobic; RES, respiration-permissive condition.
