# Supplementary material for: Lactococcus lactis Mutants Obtained From Laboratory Evolution Showed Elevated Vitamin K2 Content and Enhanced Resistance to Oxidative Stress
Source: Front Microbiol. 2021 Oct 14;12:746770. doi: 10.3389/fmicb.2021.746770 (PMC8551700; doi:10.3389/fmicb.2021.746770)
Supplement: Supplementary file 2 [file Image_2.pdf]

Supplementary materials – Figures

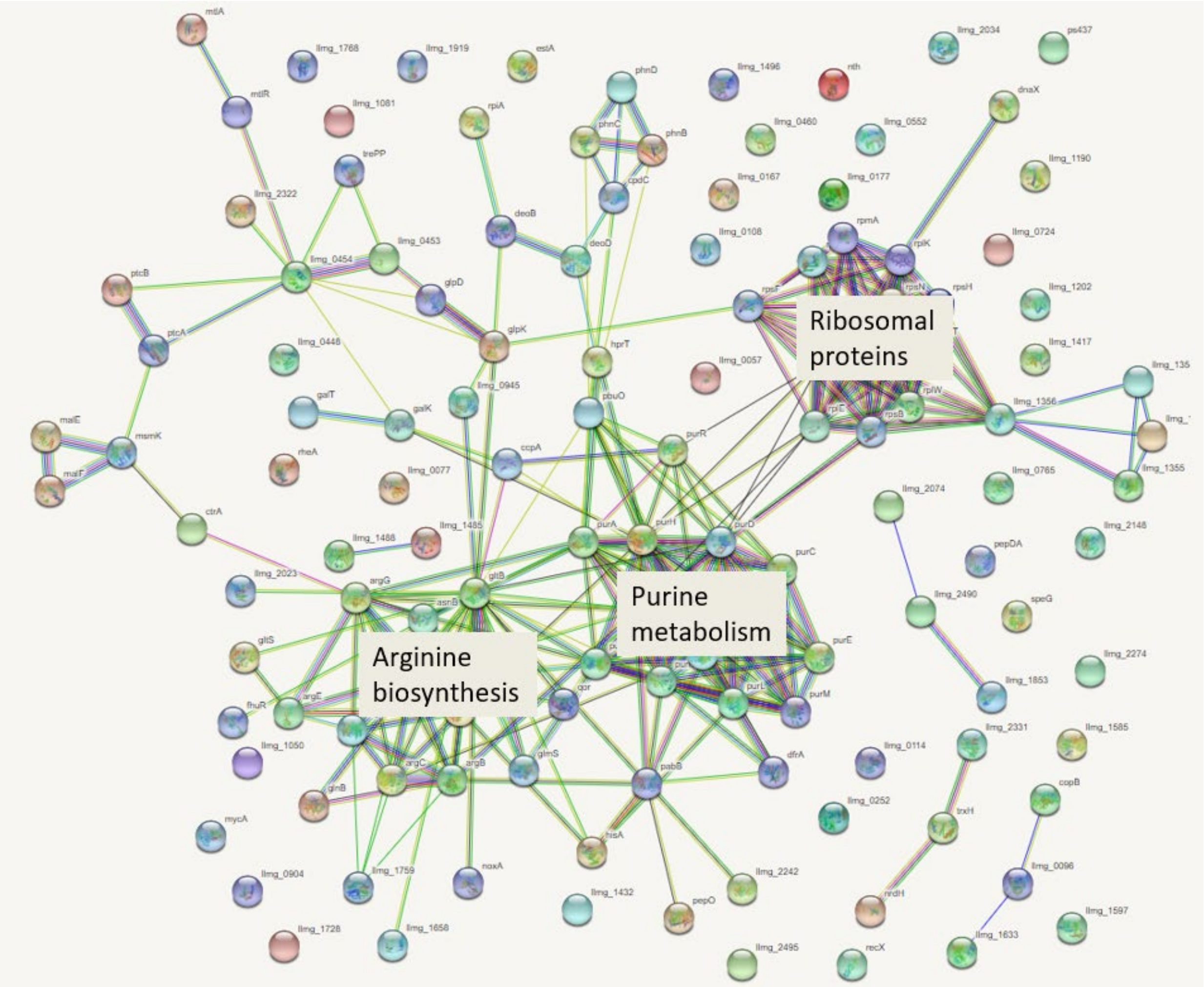

Figure S2. Protein network/associations of the 129 proteins differentially produced in Evo3 versus MG1363 under all conditions.
