# Supplementary material for: Lactococcus lactis Mutants Obtained From Laboratory Evolution Showed Elevated Vitamin K2 Content and Enhanced Resistance to Oxidative Stress
Source: Front Microbiol. 2021 Oct 14;12:746770. doi: 10.3389/fmicb.2021.746770 (PMC8551700; doi:10.3389/fmicb.2021.746770)
Supplement: Supplementary file 3 [file Image_3.pdf]

Supplementary materials – Figures

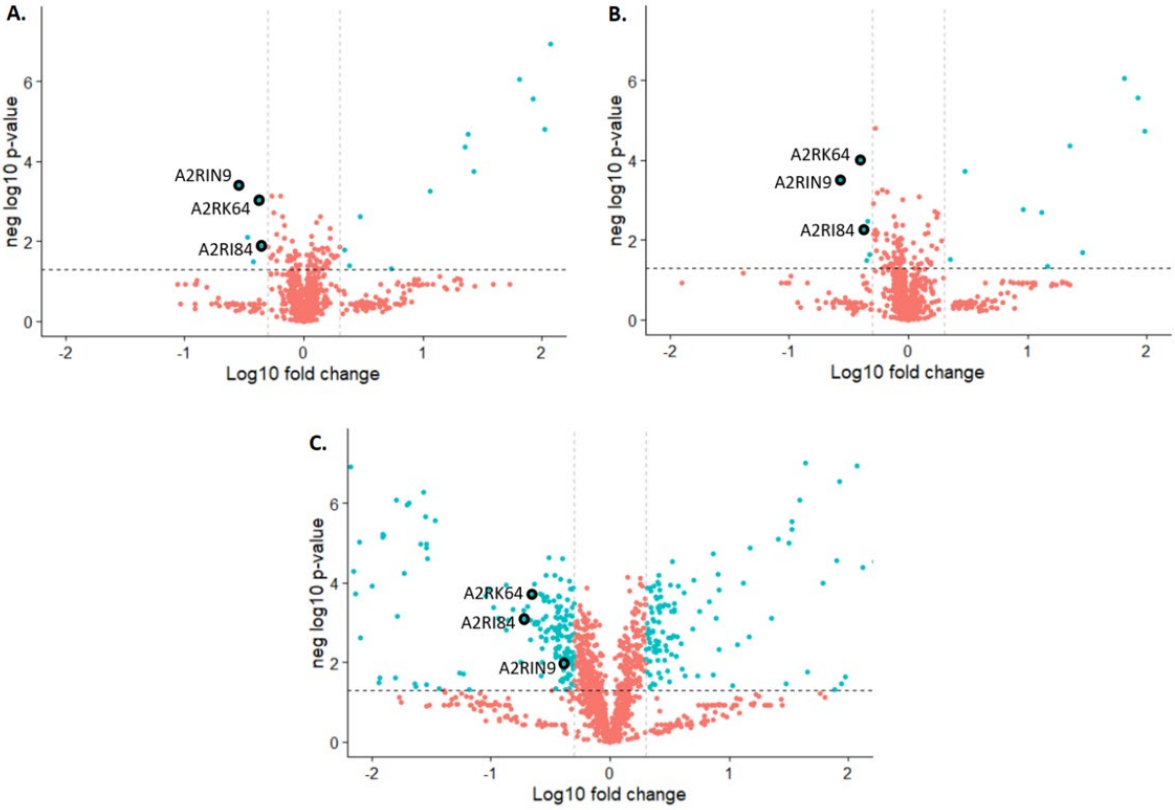

Figure S3. Volcano plots of proteome changes between evolved strains and MG1363 under aerobic condition. A) MG1363 vs. Evo1; B) MG1363 vs. Evo2; C) MG1363 vs. Evo3. In all three comparisons, proteins A2RK64, A2RI84 and A2RIN9 were significantly ($p \leq 0.05$, fold change ≥ 2) overproduced in the evolved strains compared to MG1363 (i.e. underproduced in MG1363 compared to evolved strains), as indicated by black circles. Data from three independent experiments.
