# Supplementary material for: Lactococcus lactis Mutants Obtained From Laboratory Evolution Showed Elevated Vitamin K2 Content and Enhanced Resistance to Oxidative Stress
Source: Front Microbiol. 2021 Oct 14;12:746770. doi: 10.3389/fmicb.2021.746770 (PMC8551700; doi:10.3389/fmicb.2021.746770)
Supplement: Supplementary file 4 [file Image_4.pdf]

Supplementary materials - Figures

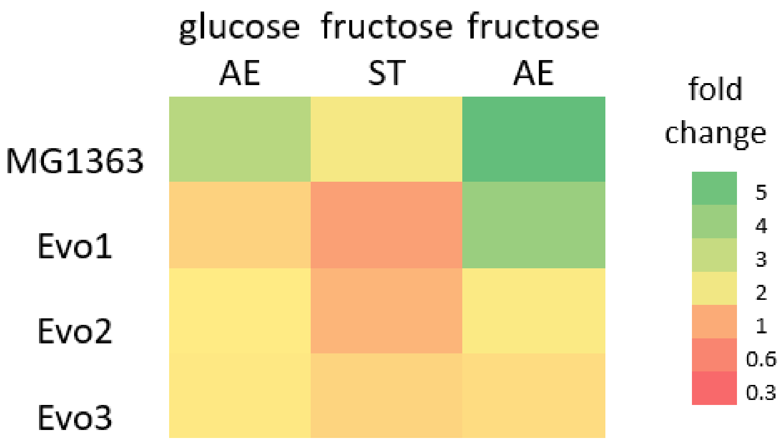

Figure S4. Changes in the total vitamin K2 production under varied conditions in respective strains. MG1363 and evolved strains were cultivated in M17 media supplemented with 0.5% (w/v) indicated carbon source (glucose or fructose), under indicated conditions (ST or AE), at 30°C for 48 h. Fold changes were calculated taking values of "glucose ST" condition as the reference of respective strains.
