# Supplementary material for: Lactococcus lactis Mutants Obtained From Laboratory Evolution Showed Elevated Vitamin K2 Content and Enhanced Resistance to Oxidative Stress
Source: Front Microbiol. 2021 Oct 14;12:746770. doi: 10.3389/fmicb.2021.746770 (PMC8551700; doi:10.3389/fmicb.2021.746770)
Supplement: Supplementary file 5 [file Table_1.docx]

**Supplementary materials - Tables**

**Table S1. Quantity (Log LFQ intensity) of proteins encoded by mutated genes in evolved strains compared to strain MG1363 under various cultivation conditions.** Values are average from samples collected from 3 independent experiments, SEM values are shown in brackets. Detection limit in Log LFQ intensity: 6.3; ND = not detected, and “-” indicates that SEM values are not applicable in this case.

| Gene name (locus) | Protein ID | ST | | | | AE | | | | RES | | | |
| --- | --- | --- | --- | --- | --- | --- | --- | --- | --- | --- | --- | --- | --- |
|  |  | **MG1363** | **Evo1** | **Evo2** | **Evo3** | **MG1363** | **Evo1** | **Evo2** | **Evo3** | **MG1363** | **Evo1** | **Evo2** | **Evo3** |
| rplA (llmg_2276) | A2RNF3 | 10.78 | 10.81 | 10.85 | 10.61 | 10.84 | 10.85 | 10.83 | 10.52 | 10.76 | 10.78 | 10.77 | 10.46 |
|  |  | (0.03) | (0.01) | (0.05) | (0.03) | (0.03) | (0.03) | (0.03) | (0.03) | (0.04) | (0.02) | (0.03) | (0.07) |
| ftsL (llmg_1680) | A2RLT3 | ND | ND | ND | ND | ND | ND | ND | ND | ND | ND | ND | ND |
|  |  | - | - | - | - | - | - | - | - | - | - | - | - |
| purR (llmg_2551) | P0A400 | 9.28 | 9.27 | 9.32 | 8.12 | 9.22 | 9.17 | 9.22 | 7.70 | 9.28 | 9.26 | 9.27 | 8.35 |
|  |  | (0.01) | (0.04) | (0.04) | (0.15) | (0.04) | (0.03) | (0.04) | (0.03) | (0.03) | (0.02) | (0.04) | (0.07) |
| llmg_0907 | A2RJP8 | 8.77 | 8.84 | 8.87 | 8.95 | 8.82 | 8.88 | 8.86 | 8.91 | 8.85 | 8.90 | 8.93 | 9.00 |
|  |  | (0.03) | (0.02) | (0.02) | (0.02) | (0.01) | (0.02) | (0.05) | (0.07) | (0.01) | (0.01) | (0.03) | (0.03) |
| ldh (llmg_1120) | A2RKA4 | 10.92 | 10.93 | 10.89 | 11.15 | 10.76 | 10.74 | 10.76 | 10.91 | 10.65 | 10.68 | 10.67 | 10.95 |
|  |  | (0.01) | (0.03) | (0.04) | (0.01) | (0.01) | (0.02) | (0.02) | (0.02) | (0.00) | (0.00) | (0.01) | (0.01) |
| ps435 (llmg_2107) | A2RMY7 | ND | ND | ND | ND | ND | ND | ND | ND | ND | ND | ND | ND |
|  |  | - | - | - | - | - | - | - | - | - | - | - | - |
| gapB (llmg_2539) | A2RP55 | 11.46 | 11.51 | 11.48 | 11.74 | 11.51 | 11.57 | 11.56 | 11.63 | 11.46 | 11.50 | 11.49 | 11.84 |
|  |  | (0.04) | (0.02) | (0.04) | (0.09) | (0.03) | (0.03) | (0.02) | (0.05) | (0.02) | (0.03) | (0.04) | (0.04) |
