# Supplementary material for: Lactococcus lactis Mutants Obtained From Laboratory Evolution Showed Elevated Vitamin K2 Content and Enhanced Resistance to Oxidative Stress
Source: Front Microbiol. 2021 Oct 14;12:746770. doi: 10.3389/fmicb.2021.746770 (PMC8551700; doi:10.3389/fmicb.2021.746770)
Supplement: Supplementary file 6 [file Table_2.docx]

**Supplementary materials - Tables**

**Table S2. Quantity (Log LFQ intensity) of proteins involved in the vitamin K2 synthesis pathway (kegg pathway llm00130) in strain MG1363 and evolved strains under various cultivation conditions.** Values are average from samples collected from 3 independent experiments, SEM values are shown in brackets. Detection limit in Log LFQ intensity: 6.3; ND = not detected, and “-” indicates that SEM values are not applicable in this case.

| Gene name (locus) | Protein ID | ST | | | | AE | | | | RES | | | |
| --- | --- | --- | --- | --- | --- | --- | --- | --- | --- | --- | --- | --- | --- |
|  |  | **MG1363** | **Evo1** | **Evo2** | **Evo3** | **MG1363** | **Evo1** | **Evo2** | **Evo3** | **MG1363** | **Evo1** | **Evo2** | **Evo3** |
| menF (llmg_1828) | A2RM72 | 8.32 | 8.24 | 8.32 | 8.14 | 8.18 | 8.28 | 8.26 | 7.52 | 8.07 | 8.20 | 8.06 | 8.00 |
|  |  | (0.03) | (0.04) | (0.07) | (0.07) | (0.03) | (0.04) | (0.04) | (0.61) | (0.04) | (0.03) | (0.03) | (0.05) |
| menD (llmg_1829) | A2RM73 | 9.03 | 8.96 | 8.96 | 8.94 | 9.00 | 8.96 | 9.00 | 8.99 | 8.88 | 8.88 | 8.94 | 8.86 |
|  |  | (0.02) | (0.01) | (0.01) | (0.02) | (0.03) | (0.02) | (0.01) | (0.05) | (0.01) | (0.03) | (0.02) | (0.04) |
| menX (llmg_1830) | A2RM74 | ND | ND | ND | ND | ND | ND | ND | ND | ND | ND | ND | ND |
|  |  | - | - | - | - | - | - | - | - | - | - | - | - |
| menC (llmg_1833) | A2RM77 | 8.29 | 8.19 | 8.25 | 8.40 | 8.11 | 8.29 | 8.25 | 8.40 | 8.23 | 8.25 | 8.25 | 8.33 |
|  |  | (0.06) | (0.02) | (0.07) | (0.03) | (0.03) | (0.00) | (0.04) | (0.05) | (0.04) | (0.05) | (0.06) | (0.08) |
| menE (llmg_1832) | A2RM76 | 8.55 | 8.44 | 8.54 | 8.72 | 8.48 | 8.40 | 8.38 | 8.71 | 8.48 | 8.50 | 8.47 | 8.61 |
|  |  | (0.03) | (0.02) | (0.01) | (0.01) | (0.02) | (0.09) | (0.04) | (0.06) | (0.02) | (0.02) | (0.03) | (0.05) |
| menB (llmg_1831) | A2RM75 | 9.87 | 9.83 | 9.86 | 9.74 | 9.98 | 10.01 | 9.95 | 9.79 | 9.98 | 9.97 | 10.01 | 9.95 |
|  |  | (0.02) | (0.04) | (0.04) | (0.06) | (0.01) | (0.01) | (0.04) | (0.06) | (0.01) | (0.00) | (0.01) | (0.04) |
| menA (llmg_0197) | A2RHR6 | ND | ND | ND | ND | ND | ND | ND | ND | ND | ND | ND | ND |
|  |  | - | - | - | - | - | - | - | - | - | - | - | - |
| menG (llmg_0753) | A2RJA5 | 8.74 | 8.71 | 8.81 | 8.89 | 8.81 | 8.79 | 8.77 | 8.96 | 8.73 | 8.70 | 8.74 | 8.84 |
|  |  | (0.03) | (0.03) | (0.02) | (0.05) | (0.04) | (0.05) | (0.02) | (0.05) | (0.01) | (0.01) | (0.01) | (0.04) |
| llmg_0196 | A2RHR5 | 8.84 | 8.87 | 8.83 | 8.93 | 8.88 | 8.89 | 8.88 | 8.95 | 8.81 | 8.85 | 8.80 | 8.95 |
|  |  | (0.02) | (0.02) | (0.01) | (0.01) | (0.02) | (0.01) | (0.01) | (0.03) | (0.01) | (0.01) | (0.02) | (0.03) |
| ispB (llmg_1110) | A2RK94 | 8.61 | 8.57 | 8.62 | 8.43 | 8.50 | 8.42 | 8.52 | 8.52 | 8.38 | 8.37 | 8.38 | 8.33 |
|  |  | (0.03) | (0.04) | (0.03) | (0.14) | (0.04) | (0.08) | (0.05) | (0.07) | (0.05) | (0.05) | (0.10) | (0.10) |
| GerCA (llmg_1111) | A2RK95 | ND | ND | ND | ND | ND | ND | ND | ND | ND | ND | ND | ND |
|  |  | - | - | - | - | - | - | - | - | - | - | - | - |
| mvk (llmg_0425) | A2RID4 | 9.27 | 9.22 | 9.23 | 9.14 | 9.23 | 9.20 | 9.21 | 9.21 | 9.09 | 9.09 | 9.08 | 9.12 |
|  |  | (0.03) | (0.04) | (0.04) | (0.02) | (0.00) | (0.03) | (0.01) | (0.05) | (0.02) | (0.01) | (0.01) | (0.05) |
