# Supplementary material for: Lactococcus lactis Mutants Obtained From Laboratory Evolution Showed Elevated Vitamin K2 Content and Enhanced Resistance to Oxidative Stress
Source: Front Microbiol. 2021 Oct 14;12:746770. doi: 10.3389/fmicb.2021.746770 (PMC8551700; doi:10.3389/fmicb.2021.746770)
Supplement: Supplementary file 7 [file Table_3.docx]

**Supplementary materials - Tables**

**Table S3. Quantity (Log LFQ intensity) of NADH dehydrogenases in strain MG1363 and evolved strains under various cultivation conditions.** Values are average from samples collected from 3 independent experiments, SEM values are shown in brackets. Detection limit in Log LFQ intensity: 6.3; ND = not detected, and “-” indicates that SEM values are not applicable in this case.

| Gene name (locus) | Protein ID | ST | | | | AE | | | | RES | | | |
| --- | --- | --- | --- | --- | --- | --- | --- | --- | --- | --- | --- | --- | --- |
|  |  | **MG1363** | **Evo1** | **Evo2** | **Evo3** | **MG1363** | **Evo1** | **Evo2** | **Evo3** | **MG1363** | **Evo1** | **Evo2** | **Evo3** |
| noxA  (llmg_1735) | A2RLY1 | 9.79 | 9.76 | 9.79 | 8.92 | 9.86 | 9.82 | 9.84 | 9.03 | 9.62 | 9.64 | 9.62 | 9.01 |
|  |  | (0.03) | (0.05) | (0.05) | (0.05) | (0.01) | (0.02) | (0.02) | (0.07) | (0.00) | (0.02) | (0.02) | (0.06) |
| noxB  (llmg_1734) | A2RLY0 | 10.17 | 10.17 | 10.18 | 9.93 | 10.14 | 10.14 | 10.16 | 9.96 | 10.02 | 10.03 | 10.03 | 9.84 |
|  |  | (0.04) | (0.02) | (0.07) | (0.04) | (0.02) | (0.02) | (0.02) | (0.04) | (0.03) | (0.02) | (0.01) | (0.03) |
| noxC  (llmg_1770) | A2RM15 | 9.11 | 9.03 | 9.06 | 9.26 | 9.09 | 9.08 | 9.05 | 9.14 | 9.18 | 9.18 | 9.15 | 9.18 |
|  |  | (0.04) | (0.05) | (0.05) | (0.07) | (0.02) | (0.02) | (0.08) | (0.02) | (0.01) | (0.01) | (0.02) | (0.05) |
| noxE  (llmg_0408) | A2RIB7 | 10.11 | 10.12 | 10.11 | 9.92 | 10.41 | 10.35 | 10.37 | 10.20 | 9.76 | 9.81 | 9.83 | 9.90 |
|  |  | (0.05) | (0.07) | (0.06) | (0.05) | (0.02) | (0.00) | (0.01) | (0.05) | (0.02) | (0.01) | (0.03) | (0.03) |
