# Supplementary material for: Lactococcus lactis Mutants Obtained From Laboratory Evolution Showed Elevated Vitamin K2 Content and Enhanced Resistance to Oxidative Stress
Source: Front Microbiol. 2021 Oct 14;12:746770. doi: 10.3389/fmicb.2021.746770 (PMC8551700; doi:10.3389/fmicb.2021.746770)
Supplement: Supplementary file 8 [file Table_4.docx]

**Supplementary materials - Tables**

**Table S4. Quantity (Log LFQ intensity) of proteins involved in pyruvate metabolism in strain MG1363 and evolved strains under various cultivation conditions.** Values are average from samples collected from 3 independent experiments, SEM values are shown in brackets. Detection limit in Log LFQ intensity: 6.3; ND = not detected, and “-” indicates that SEM values are not applicable in this case.

| Gene name (locus) | Protein ID | ST | | | | AE | | | | RES | | | |
| --- | --- | --- | --- | --- | --- | --- | --- | --- | --- | --- | --- | --- | --- |
|  |  | **MG1363** | **Evo1** | **Evo2** | **Evo3** | **MG1363** | **Evo1** | **Evo2** | **Evo3** | **MG1363** | **Evo1** | **Evo2** | **Evo3** |
| pfl  (llmg_0629) | O32799 | 10.37 | 10.27 | 10.31 | 10.28 | 9.88 | 9.87 | 9.91 | 10.11 | 10.39 | 10.50 | 10.53 | 10.48 |
|  |  | (0.03) | (0.04) | (0.02) | (0.04) | (0.02) | (0.01) | (0.00) | (0.03) | (0.04) | (0.02) | (0.01) | (0.03) |
| pflA  (llmg_1997) | A2RMN3 | 8.93 | 8.93 | 8.98 | 9.23 | 7.39 | ND | 7.36 | 9.01 | 8.28 | 8.41 | 8.47 | 9.15 |
|  |  | (0.03) | (0.03) | (0.02) | (0.01) | (0.55) | - | (0.54) | (0.04) | (0.04) | (0.06) | (0.08) | (0.04) |
| ldhB  (llmg_0392) | P0CI34 | 6.76 | 7.28 | 7.27 | ND | 6.77 | 6.77 | 7.27 | 6.71 | 6.69 | 6.76 | ND | ND |
|  |  | (0.45) | (0.49) | (0.48) | - | (0.47) | (0.47) | (0.49) | (0.41) | (0.39) | (0.46) | - | - |
| ldhX  (llmg_1429) | A2RL45 | 8.54 | 8.49 | 8.45 | 8.01 | 8.06 | 7.84 | 7.91 | 7.91 | 7.98 | 7.96 | 7.96 | 8.00 |
|  |  | (0.06) | (0.07) | (0.06) | (0.06) | (0.01) | (0.04) | (0.07) | (0.02) | (0.02) | (0.02) | (0.08) | (0.02) |
| ldh | A2RKA4 | 10.92 | 10.93 | 10.89 | 11.15 | 10.76 | 10.74 | 10.76 | 10.91 | 10.65 | 10.68 | 10.67 | 10.95 |
| (llmg_1120) |  | (0.01) | (0.03) | (0.04) | (0.01) | (0.01) | (0.02) | (0.02) | (0.02) | (0.00) | (0.00) | (0.01) | (0.01) |
| pdhD  (llmg_0071) | A2RHE1 | 10.42 | 10.41 | 10.40 | 10.37 | 10.56 | 10.59 | 10.60 | 10.36 | 10.69 | 10.69 | 10.70 | 10.47 |
|  |  | (0.02) | (0.02) | (0.01) | (0.02) | (0.00) | (0.02) | (0.02) | (0.02) | (0.01) | (0.01) | (0.01) | (0.03) |
| pdhC  (llmg_0072) | A2RHE2 | 10.61 | 10.60 | 10.58 | 10.58 | 10.81 | 10.81 | 10.85 | 10.55 | 10.91 | 10.93 | 10.94 | 10.62 |
|  |  | (0.04) | (0.03) | (0.03) | (0.07) | (0.01) | (0.02) | (0.01) | (0.01) | (0.02) | (0.00) | (0.01) | (0.02) |
| pdhB  (llmg_0073) | A2RHE3 | 10.70 | 10.69 | 10.67 | 10.79 | 10.81 | 10.83 | 10.85 | 10.67 | 10.91 | 10.94 | 10.92 | 10.78 |
|  |  | (0.03) | (0.02) | (0.03) | (0.06) | (0.03) | (0.03) | (0.02) | (0.01) | (0.01) | (0.02) | (0.01) | (0.03) |
| pdhA  (llmg_0074) | A2RHE4 | 10.28 | 10.28 | 10.27 | 10.24 | 10.42 | 10.42 | 10.41 | 10.15 | 10.56 | 10.54 | 10.59 | 10.29 |
|  |  | (0.02) | (0.04) | (0.02) | (0.03) | (0.01) | (0.01) | (0.01) | (0.02) | (0.02) | (0.02) | (0.02) | (0.05) |
